# Changes in thyroid function and evolution of subclinical thyroid disease in older men

**DOI:** 10.1111/cen.14997

**Published:** 2023-12-07

**Authors:** Stephanie Y. Tan, S. A. Paul Chubb, Leon Flicker, Osvaldo P. Almeida, Jonathan Golledge, Graeme J. Hankey, Bu B. Yeap

**Affiliations:** ^1^ Medical School University of Western Australia Perth WA Australia; ^2^ Clinical Biochemistry Department, PathWest Laboratory Medicine Fiona Stanley Hospital Perth WA Australia; ^3^ WA Centre for Health & Ageing University of Western Australia Perth WA Australia; ^4^ Queensland Research Centre for Peripheral Vascular Disease James Cook University Townsville QLD Australia; ^5^ Department of Vascular and Endovascular Surgery Townsville Hospital Townsville QLD Australia; ^6^ Perron Institute for Neurological and Translational Science Perth WA Australia; ^7^ Department of Endocrinology and Diabetes Fiona Stanley Hospital Perth WA Australia

**Keywords:** ageing, free thyroxine, hypothyroidism, men, subclinical hyperthyroidism, subclinical hypothyroidism, thyroid‐stimulating hormone

## Abstract

**Objective:**

Prevalence of subclinical thyroid disease increases with age, but optimal detection and surveillance strategies remain unclear particularly for older men. We aimed to assess thyroid stimulating hormone (TSH) and free thyroxine (FT4) concentrations and their longitudinal changes, to determine the prevalence and incidence of subclinical thyroid dysfunction in older men.

**Design, Participants and Measurements:**

Longitudinal study of 994 community‐dwelling men aged ≥70 years without known or current thyroid disease, with TSH and FT4 concentrations assessed at baseline and follow‐up (after 8.7 ± 0.9 years). Factors associated with incident subclinical thyroid dysfunction were examined by logistic regression and receiver operating characteristic analyses.

**Results:**

At baseline, 85 men (8.6%) had subclinical hypothyroidism and 10 (1.0%) subclinical hyperthyroidism. Among 899 men euthyroid at baseline (mean age 75.0 ± 3.0 years), 713 (79.3%) remained euthyroid, 180 (20.0%) developed subclinical/overt hypothyroidism, and 6 (0.7%) subclinical/overt hyperthyroidism. Change in TSH correlated with baseline TSH (r = .16, *p* < .05). Change in FT4 correlated inversely with baseline FT4 (r = −0.35, *p* < .05). Only higher age and baseline TSH predicted progression from euthyroid to subclinical/overt hypothyroidism (fully‐adjusted odds ratio [OR] per year=1.09, 95% confidence interval [CI] = 1.02‐1.17, *p* = .006; per 2.7‐fold increase in TSH OR = 65.4, CI = 31.9‐134, *p* < .001). Baseline TSH concentration ≥2.34 mIU/L had 76% sensitivity and 77% specificity for predicting development of subclinical/overt hypothyroidism.

**Conclusions:**

In older men TSH concentration increased over time, while FT4 concentration showed little change. Subclinical or overt hypothyroidism evolved in one fifth of initially euthyroid men, age and higher baseline TSH predicted this outcome. Increased surveillance for thyroid dysfunction may be justified in older men, especially those with high‐normal TSH.

## 1 INTRODUCTION

Subclinical hyperthyroidism is characterised by low serum thyroid‐stimulating hormone (TSH) but normal thyroid hormone concentrations, without clinically apparent symptoms and signs of hyperthyroidism.^1^, ^2^ Its prevalence in community‐dwelling adults is around 2%–3%.^2^ Subclinical hypothyroidism is defined by raised TSH and normal thyroid hormone concentration, without clinical indicators of hypothyroidism.^3^ Subclinical hypothyroidism has a prevalence of around 2%–4%.^2^ Ageing is accompanied by an increased prevalence of both subclinical hyperthyroidism and subclinical hypothyroidism.^4^ Subclinical hyperthyroidism has been associated with weight loss, osteoporosis and atrial fibrillation.^5^ Subclinical hypothyroidism has been associated with adverse health outcomes, including increased risk of heart failure, coronary artery disease events and coronary heart disease mortality.^6^ As such, there has been an ongoing debate about the possible adverse health complications, screening recommendations and treatment advice for thyroid disease.

Higher serum TSH concentrations are commonly found in older adults, suggesting an age‐related change in hypothalamic‐pituitary‐thyroid axis function.^7^ Cross‐sectional studies have shown that older people on average have higher TSH than younger people.^8^, ^9^ There have been few longitudinal studies examining changes in serum TSH and free thyroxine (FT4) concentrations via serial blood sampling in older populations and the results for both markers have been inconsistent.^10^, ^11^, ^12^ Furthermore, those studies included limited numbers of older men. Therefore, it remains unclear whether the changes in thyroid function seen with ageing reflect a general shift in thyroid physiology, or evolution of thyroid disease, particularly amongst the expanding demographic of older men.

We aimed to assess the prevalence and incidence of subclinical thyroid disease in older men without a history of known thyroid disease, analyse the longitudinal trajectories of TSH and FT4 in this population, and examine age and other clinical and biochemical factors associated with the development of subclinical or overt hypothyroidism. We aimed to test the hypothesis that amongst men who remained euthyroid, both TSH and FT4 remained stable during follow‐up, thus providing evidence against a change in thyroid physiology with age.

## 2 PARTICIPANTS AND METHODS

### 2.1 Study population

**Figure 1:**
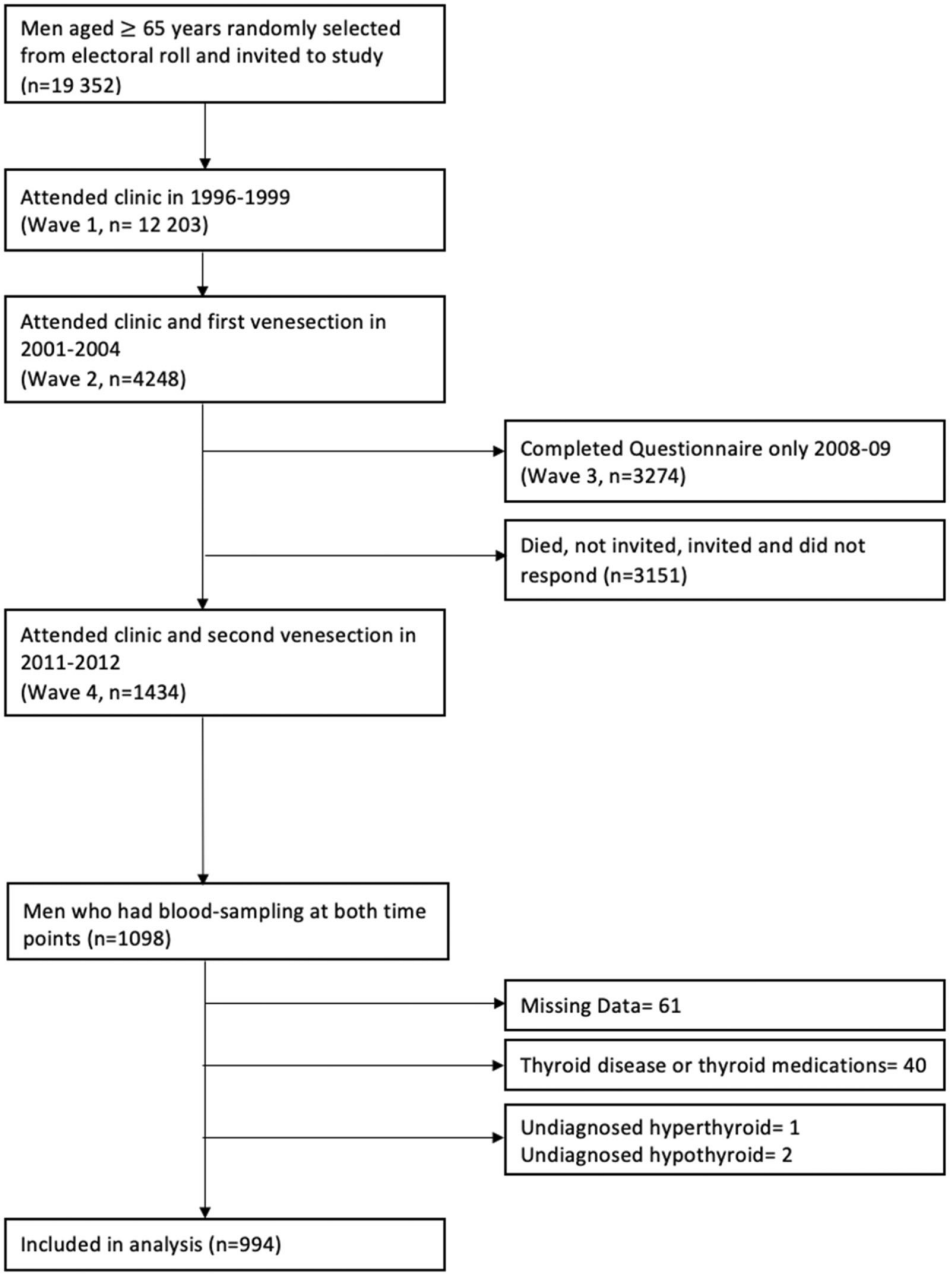
Participant flow chart showing derivation of the study cohort. There were four waves of data collection, and during two of those (Waves 2 and 4) blood samples were collected. The analysis population for this study comprises men with blood samples collected (and hence available for analysis of thyroid‐stimulating hormone and free thyroxine concentrations) at both timepoints (Wave 2 and Wave 4), after excluding those with missing data or known or current thyroid disease.

The Health In Men Study (HIMS) is a prospective cohort study.^13^ Here, 19,352 men aged >65 years from Perth, Western Australia, were randomly selected from the electoral roll (enrolment to vote is compulsory for all Australian adults) and invited to join a screening trial for abdominal aortic aneurysms. Then, 12,203 men accepted and were assessed in 1996–1999 (Wave 1). In 2001–2004 (Wave 2), 4248 of these men (then aged 70–89 years) returned for reassessment, physical examination and venesection. A questionnaire survey was conducted in 2008–2009 (Wave 3). In 2011–2012 (Wave 4), 1434 men returned for reassessment and a second venesection. This analysis cohort comprises the 1098 men who had blood sampling in both 2001–2004 and 2011–2012. We excluded 40 men with recorded thyroid disease or using thyroid‐related medications and 61 men with missing data. One man with previously undiagnosed hyperthyroidism (TSH < 0.4 mU/L and FT4 > 23 pmol/L) and two with undiagnosed hypothyroidism (TSH > 4.0 mU/L and FT4 < 10 pmol/L) were also excluded, leaving 994 men (Figure 1). The University of Western Australia Human Research Ethics Committee approved the study and all men gave written informed consent.

### 2.2 Demographic variables and physical measurements

Demographic and lifestyle data, past medical history and medications usage was recorded via questionnaire in 2001–2004 and 2011–2012. Height, waist circumference, weight, systolic blood pressure and diastolic blood pressure were measured. Hypertension was categorised as blood pressure ≥ 140/90 mmHg or antihypertensive medication use. Dyslipidaemia was categorised as fasting high‐density lipoprotein cholesterol (HDL) < 0.9 mmol/L, low‐density lipoprotein cholesterol (LDL) ≥ 3.4 mmol/L, triglycerides ≥ 1.8 mmol/L or total cholesterol ≥ 5.5 mmol/L, or use of lipid‐lowering therapy. Diabetes was categorised as fasting blood glucose ≥ 7.0 mmol/L, nonfasting blood glucose ≥ 11.1 mmol/L, HbA1c > 6.5% (measured in 2011–2012 only) or use of blood glucose‐lowering medications. Global perception of health was assessed at both timepoints via the question ‘In general, would you say your health is…?’. Responses were: ‘excellent’, ‘very good’, ‘good’, ‘fair’ and ‘poor’.^14^

### 2.3 Identification of thyroid disease

Questionnaire data revealed men who had previously been diagnosed with thyroid diseases (such as hyperthyroidism, hypothyroidism, thyroid tumours, thyroid nodules) and men who were taking thyroid related medications. International Classification of Disease 10th Edition codes for iodine deficiency (E00, E01, E02), established hypothyroidism or hyperthyroidism (E03, E05), thyroiditis (E05), nontoxic goitre (E04), other specified disorders of thyroid (E07,8) were used to identify men with thyroid disease, as were ICD‐9 codes 240.x‐246.x.

### 2.4 Biochemical assays

Laboratory assays were performed on aliquots of serum and plasma prepared immediately after blood sampling and stored at −80°C until time of assay. Serum TSH and FT4 were measured in samples collected in 2001–2004 and 2011–2012, and anti‐thyroperoxidase antibody (TPO) concentrations measured in samples from 2011 to 2012 only (Elecsys 2010 immunoanalyser, Roche Diagnostics Australia). Between‐run imprecision values (coefficient of variation, %CV) for quality control samples were: for TSH, 3.1% and 3.2% at 1.5 and 8.7 mIU/L, respectively; for FT4, 6.6% and 7.1% at 16 (1.2) and 40 pmol/L (3.1 ng/dL), respectively; and for anti‐TPO, 12.4% and 8.5% at 33 and 83 U/L, respectively. Reference intervals for these assays based on local clinical laboratory protocols were TSH: 0.4–4.0 mIU/L, FT4: 10–23 pmol/L (0.78–1.79 ng/dL) and anti‐TPO < 35 U/L. To convert FT4 in pmol/L to ng/dL, divide by 12.87.

Fasting total and HDL cholesterol and total triglycerides were assayed (Hitachi 917 analyser, Roche Diagnostics Australia). LDL was estimated using the Friedewald equation.^15^ For total cholesterol, %CV was 2.3% and 2.1% at 3.2 and 6.7 mmol/L, respectively; for HDL, 2.4% and 2.5% at 0.8 and 1.7 mmol/L, respectively; and for total triglycerides, 4.8% and 2.4% at 0.9 and 2.0 mmol/L, respectively.

### 2.5 Definition of subclinical and overt hyper‐and hypothyroidism

Based on accepted reference ranges for the assays used, men with TSH < 0.4 mU/L and FT4 > 23 pmol/L were classified as having overt hyperthyroidism. Men with TSH < 0.4 mU/L and FT4  10–23 pmol/L were classified as having subclinical hyperthyroidism. Men with TSH > 4.0 mU/L and FT4  10–23 pmol/L were classified as having subclinical hypothyroidism. Men with TSH > 4.0 mU/L and FT4 < 10 pmol/L were classified as having overt hypothyroidism. At follow‐up, men taking thyroxine were also categorised as having developed overt hypothyroidism.

### 2.6 Statistical analysis

Continuous data were reported as mean values and standard deviations whilst categorical data were reported as numbers and percentages (%). TSH data were skewed to the right and were log transformed for further analysis. Independent t‐tests were used to compare differences in means between individual variables and paired t‐tests were used to evaluate changes in FT4 and TSH between baseline and follow‐up. Analysis of variance and *χ* ^2^ tests were used to compare differences between groups. Tukey's posthoc analysis was then applied to compare means of variables. Pearson's coefficients were used to analyse the relationship between continuous variables. Multivariate regression was used to assess factors associated with development of overt or subclinical hypothyroidism in men who were euthyroid at baseline and included the following variables: TSH, FT4, body mass index (BMI), age, smoking status, hypertension, dyslipidaemia, cancer, cardiovascular disease, diabetes, dementia, depression, lipid lowering medications, antihypertensives, glucose‐lowering medications, cholesterol, HDL, LDL and triglycerides. Receiver operating characteristic (ROC) and probability analyses were performed to identify thresholds of TSH in euthyroid men, which were predictive of developing subclinical or overt hypothyroidism at follow‐up. A *p* < .05 was considered significant. All statistical analysis was performed using STATA 17.0.

## 3 RESULTS

### 3.1 Characteristics of study participants at baseline

The baseline characteristics of the 994 participants (Wave 2) are summarised, stratified according to euthyroid, subclinical hypothyroid and subclinical hyperthyroid status (Table 1). Most men (90%) were euthyroid, 8.6% had subclinical hypothyroidism and 1% had subclinical hyperthyroidism. Age, smoking status, prevalence of diabetes, cardiovascular disease, cancer, dementia and depression, medications usage, blood pressure and lipid profile were similar across all three groups. Around half the men self‐rated their health as excellent or very good. As expected, TSH and FT4 concentrations differed across groups.

**Table 1:** Baseline characteristics of the study cohort (Wave 2) stratified into men who were euthyroid and those categorised as having subclinical hypothyroidism and subclinical hyperthyroidism.

	Euthyroid	Subclinical hypothyroidism	Subclinical hyperthyroidism	*p*
*N*	899	85	10	
Age (years)	75.0 ± 3.0	75.5 ± 3.4	74.7 ± 3.7	.161
Never smoker	348 (38.7)	33 (38.8)	6 (60)	.654
Ex‐smoker	518 (57.6)	50 (58.8)	4 (40)	.654
Current Smoker	33 (3.7)	2 (2.4)	0 (0)	.654
Antihypertensive medications	516 (57.4)	44 (51.8)	4 (40)	.340
Lipid‐lowering medications	345 (38.4)	31 (36.5)	3 (30)	.818
Glucose‐lowering medications	72 (8.0)	6 (7.1)	0 (0)	.620
Hypertension	620 (69.0)	62 (72.9)	7 (70)	.749
Dyslipidaemia	417 (46.4)	43 (50.6)	6 (60)	.536
Diabetes	208 (23.1)	19 (22.4)	1 (10)	.612
Cardiovascular disease	220 (24.5)	18 (21.2)	2 (20)	.758
Cancer	410 (45.6)	35 (41.1)	7 (70)	.216
Dementia	22 (2.4)	2 (2.4)	1 (10)	.315
Depression	39 (4.3)	4 (4.7)	0 (0)	.786
Thyroid disease or thyroid medications	0	0	0	
Self‐reported health				
− Excellent/very good	498 (55.4)	49 (57.6)	4 (40)	.178
− Good/fair/poor	401 (44.6)	36 (42.4)	6 (60)	
Body mass index	26.4 ± 3.1	25.9 ± 3.4	25.9 ± 3.4	.939
Systolic BP (mmHg)	144 ± 19.3	144 ± 20.1	144.5 ± 19.2	.675
Diastolic BP (mmHg)	73 ± 9.6	72 ± 10.8	74 ± 8.2	.894
Cholesterol (mmol/L)	4.9 ± 0.93	5 ± 0.92	5.3 ± 1.15	.639
High‐density lipoprotein (mmol/L)	1.3 ± 0.35	1.3 ± 0.37	1.5 ± 0.47	.811
Low‐density lipoprotein (mmol/L)	2.9 ± 0.82	2.8 ± 0.81	3.3 ± 0.93	.889
Triglycerides (mmol/L)	1.2 ± 0.75	1.3 ± 0.89	1.2 ± 0.60	.429
TSH (mIU/L)	1.91 ± 0.80	4.78 ± 1.56	0.19 ± 0.16	<.001
FT4 (pmol/L)	15.7 ± 2.1	14.6 ± 2.1	20.6 ± 2.3	<.001
Outcomes of these individuals at follow‐up (Wave 4)				
Overt hyperthyroidism	2 (0.2)	0 (0)	3 (30)	
Subclinical hyperthyroidism	4 (0.4)	1 (1.2)	0 (0)	
Euthyroid	713 (79.3)	11 (12.9)	3 (30)	
Subclinical hypothyroidism	142 (15.8)	45 (52.9)	3 (30)	
Overt hypothyroidism (on thyroxine therapy)	34 (3.8)	27 (31.8)	1 (10)	
Overt hypothyroidism (not on thyroxine therapy)	4 (0.4)	1 (1.2)	0 (0)	

Abbreviations: BP, blood pressure; FT4, free thyroxine; TSH, thyroid‐stimulating hormone.

### 3.2 Progression from euthyroid to subclinical or overt thyroid disease

Among 899 euthyroid men followed for 8.7 ± 0.9 years, 713 remained euthyroid (79.3%), 142 developed subclinical hypothyroidism (15.7%) and 38 men became overtly hypothyroid (4.2%) (Table 1). Six men developed subclinical (four) or overt hyperthyroidism (two). Of the 85 men who had subclinical hypothyroidism at baseline, 28 men became overtly hypothyroid (32.9%), 45 remained subclinical hypothyroid (52.9%) and 11 men reverted to euthyroid (12.9%). One man developed subclinical hyperthyroidism.

Very few men had subclinical hyperthyroidism (10), of these three became overtly hyperthyroid, whereas the other men reverted into either euthyroid (three), subclinical hypothyroid (three) or overt hypothyroid (one) states.

### 3.3 Longitudinal changes in TSH and FT4

**Figure 2:**
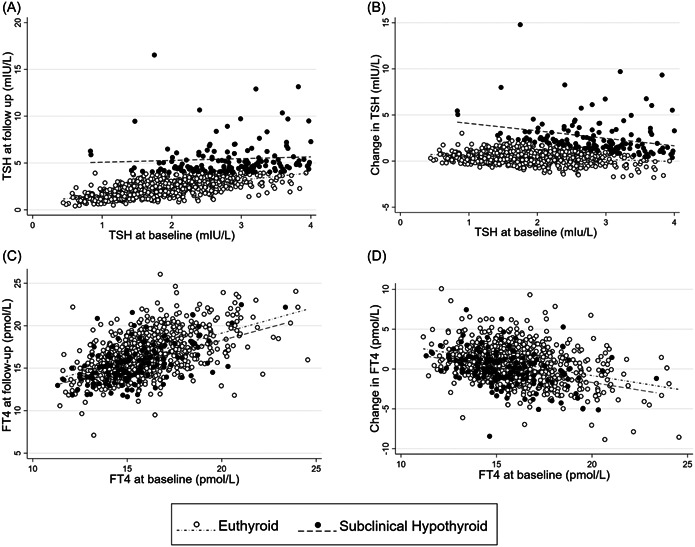
Scatterplots and regression lines showing relationships between (A) baseline thyroid‐stimulating hormone (TSH) concentrations (*x* axis) and follow‐up TSH concentrations (*y* axis); (B) baseline TSH concentrations (*x* axis) and change in TSH concentrations (*y* axis); (C) baseline free thyroxine (FT4) concentrations (*x* axis) and follow‐up FT4 concentrations (*y* axis); (D) baseline FT4 concentrations (*x* axis) and change in FT4 concentrations (*y* axis); in men who were euthyroid at baseline and remained so at follow‐up (○) and those who were euthyroid at baseline and had developed subclinical hypothyroidism at follow‐up (●).

Follow‐up TSH (2011–2012) correlated with baseline TSH (2001–2004) (Figure 2A, *r* = .61, *p* < .05). Change in TSH showed a modest correlation with baseline TSH (Figure 2B, *r* = .16, *p* < .05). The scatterplots show a single distribution of participants, with a proportion of men developing TSH > 4 mIU/L at follow‐up and thus meeting the criterion for evolution from euthyroid to subclinical hypothyroidism (Figure 2A). Most men who developed subclinical hypothyroidism had higher baseline TSH values, largely >2 mIU/L (Figure 2A,B).

Follow‐up FT4 correlated with baseline FT4 (Figure 2C, *r* = .54, *p* < .05). Change in FT4 correlated inversely with baseline FT4 (Figure 2D, *r* = −0.35, *p* < .05). Men who developed subclinical/overt hypothyroidism were not distinguishable on the basis of FT4 from men who remained euthyroid (Figure 2C,D).

### 3.4 Characteristics of study participants at follow‐up

Characteristics at follow‐up of the 899 men who were euthyroid at baseline are shown (Table 2), stratified into those who remained euthyroid and those who developed subclinical or overt hypothyroidism. Although 142 (15.8%) of the men developed subclinical hypothyroidism, 38 (4.2%) developed overt hypothyroidism, with all but four on thyroid replacement therapy. Age, smoking status, prevalence of diabetes, cardiovascular disease, cancer, dementia and depression, medications usage, BMI, blood pressure and lipid profile were similar across all three groups.

**Table 2:** Characteristics of the study cohort at follow‐up (Wave 4), categorised as men who were initially euthyroid at baseline (Wave 2) who remained euthyroid at follow‐up (Wave 4), those who were euthyroid at baseline and had subclinical hypothyroidism at follow‐up, and those who were euthyroid at baseline and had overt hypothyroidism (receiving thyroxine therapy) at follow‐up.

	Euthyroid	Subclinical hypothyroidism	Overt hypothyroidism	*p*
*N*	713	142	38	
Age (years)	83.7 ± 3.4	84.1 ± 3.5	85.0 ± 3.8	.161
Never smoker	300 (42.1)	61 (43.0)	13 (34.2)	.891
Ex‐smoker	394 (55.3)	78 (54.9)	23 (60.5)	.891
Current Smoker	19 (2.7)	3 (2.1)	2 (5.3)	.891
Antihypertensive medications	555 (77.8)	106 (74.6)	27 (71.1)	.654
Lipid‐lowering medications	272 (38.1)	95 (66.9)	27 (71.1)	.862
Glucose‐lowering medications	94 (13.2)	22 (15.5)	4 (10.5)	.622
Hypertension	509 (71.4)	97 (68.3)	25 (65.8)	.784
Dyslipidaemia	247 (34.6)	47 (33.1)	11 (28.9)	.895
Diabetes	153 (21.5)	31 (21.8)	9 (23.7)	.608
Cardiovascular disease	285 (40.0)	45 (31.7)	17 (44.7)	.260
Cancer	461 (64.7)	97 (68.3)	25 (65.8)	.349
Dementia	19 (2.7)	1 (0.7)	0	.369
Depression	72 (10.1)	12 (8.5)	5 (13.2)	.673
Thyroid disease or thyroid medications	0	0	38	
Self‐reported health				
− Excellent/very good	276 (38.7)	62 (43.7)	8 (21.1)	.053
− Good/fair/poor	430 (60.3)	76 (53.5)	29 (76.3)	
Body mass index	26.42 ± 4.12	27.38 ± 3.53	26.10 ± 4.86	.478
Systolic BP (mmHg)	144 ± 20.5	147 ± 19.29	148 ± 21.67	.506
Diastolic BP (mmHg)	73.5 ± 12.0	73.5 ± 10.11	69.5 ± 12.80	.064
Cholesterol (mmol/L)	4.2 ± 0.98	4.5 ± 0.96	4.2 ± 0.95	.260
High‐density lipoprotein (mmol/L)	1.2 ± 0.33	1.2 ± 0.29	1.2 ± 0.47	.690
Low‐density lipoprotein (mmol/L)	2.4 ± 0.83	2.7 ± 0.85	2.4 ± 0.75	.116
Triglycerides (mmol/L)	1.1 ± 0.54	1.1 ± 0.57	1.1 ± 0.51	.435
TSH (mIU/L)	2.2 ± 0.85	4.76 ± 1.91	3.30 ± 13.52	<.001
FT4 (pmol/L)	16.5 ± 2.5	15.2 ± 2.3	17.29 ± 4.04	<.001
TPO antibody positive (≥35 U/L)	21 (2.9)	7 (4.9)	6 (15.8)	<.001
TSH change (absolute)	0.4 ± 0.68*	2.08 ± 1.98* ^,^ **	0.25 ± 13.61	<.001
FT4 change (absolute)	0.65 ± 2.23*	0.05 ± 2.06** ^,^ ***	1.83 ± 3.76	<.001

Abbreviations: FT4, free thyroxine; TPO, thyroperoxidase antibody.

* *p* < .001 compared with baseline

** *p* < .05 compared with men who remained euthyroid

*** *p* = .46 compared with baseline.

Men who remained euthyroid had significant increases in both TSH and FT4 between baseline and follow‐up, whereas those who developed subclinical hypothyroidism had a significant increase in TSH, that was greater than that in the euthyroid men, but no significant change in FT4 (Table 2). Men who developed overt hypothyroidism had a higher prevalence of anti‐TPO antibodies at follow‐up than those who remained euthyroid or developed subclinical hypothyroidism.

### 3.5 Factors predicting progression from euthyroid to subclinical/overt hypothyroidism

In a multivariable model, higher age was independently associated with progression from euthyroid to subclinical/overt hypothyroidism with an odds ratio per year of 1.09, 95% confidence interval 1.02–1.17 (*p* = .006) (Table 3). A 2.72‐fold increase in TSH was associated with an over 60‐fold higher risk of progression to subclinical/overt hypothyroidism (*p* < .001). No other variables were associated with increased risk of progression from euthyroid to subclinical/overt hypothyroidism.

**Table 3:** Multivariable logistic regression analysis of factors associated with risk of developing incident subclinical or overt hypothyroidism in 899 men who were initially euthyroid (baseline at Wave 2, follow‐up at Wave 4).

	OR	95% CI	*p*
Age (years)	1.09	1.02–1.17	.006
Ex‐smoker	0.50	0.17–1.39	.184
Current smoker	0.51	0.17–1.43	.202
Hypertension	0.98	0.65–1.50	.938
Dyslipidaemia	1.33	0.78–2.26	.297
Diabetes	1.43	0.88–2.33	.153
Cardiovascular disease	1.24	0.77–2.00	.373
Cancer	0.88	0.59–1.30	.515
Dementia	4.61	0.48–44.6	.187
Depression	2.44	0.73–8.21	.149
Body mass index	1.06	0.99–1.13	.084
Cholesterol (mmol/L)	0.67	0.10–4.51	.681
High‐density lipoprotein (mmol/L)	2.16	0.29–15.8	.447
Low‐density lipoprotein (mmol/L)	1.75	0.27–11.4	.558
Triglycerides (mmol/L)	1.39	0.61–3.19	.437
TSH (lnTSH)^a^	65.4	31.9–134	<.001
FT4 (pmol/L)	0.96	0.88–1.06	.470

Abbreviations: CI, confidence interval; FT4, free thyroxine; OR, odds ratio; TSH, thyroid‐stimulating hormone.

^a^ An increase of 1 in ln(TSH) is equivalent to a 2.72‐fold increase in TSH.

### 3.6 Threshold of TSH for progression to subclinical/overt hypothyroidism

**Figure 3:**
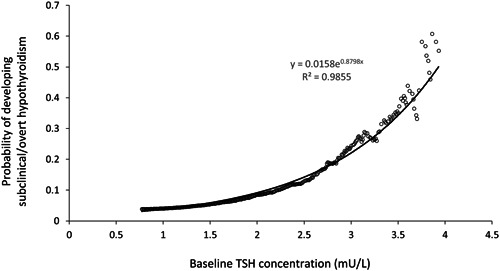
Probability of developing subclinical or overt hypothyroidism during follow‐up (*y* axis), according to baseline TSH concentrations (*x* axis), in men who were euthyroid at baseline. The fitted exponential function is Probability = 0.0158*exp(0.8798*TSH).

For the men who were euthyroid at baseline, ROC analysis demonstrated that a TSH threshold of 2.34 mU/L gave optimal sensitivity (76%) and specificity (77%) for predicting the development of subclinical or overt hypothyroidism over 8 years follow‐up. The cut‐points for 95% sensitivity and 95% specificity were 1.47 and 3.05 mU/L, respectively. The probability of developing subclinical/overt hypothyroidism based upon an individual's baseline TSH is shown (Figure 3). The risk was best modelled by an exponential function and shows risk increasing from <5% at TSH < 1 mU/L to ~50% at TSH near the upper reference limit. At TSH of 2.85 mU/L, the probability of developing subclinical/overt hypothyroidism was ≥20%.

## 4 DISCUSSION

In men aged >65 years with no history of thyroid disease, there was a generalised increase in TSH concentrations over time, which correlated with baseline TSH values. This resulted in a proportion of men crossing the threshold of TSH > 4 mIU/L and thus meeting the criterion for subclinical hypothyroidism at follow‐up. Although follow‐up FT4 concentrations correlated with baseline FT4, men with higher baseline FT4 had the smallest increases or largest decreases in FT4 over time. Most men who were euthyroid at baseline remained so at follow‐up. However, one fifth developed subclinical or overt hypothyroidism, with age and higher baseline TSH concentration markers of increased risk.

We found that the prevalence of subclinical thyroid dysfunction in the older male population was 9.6% at baseline and increased to 19.1% at follow‐up. Our findings report a higher prevalence of thyroid dysfunction compared to other population cohort studies. The Blue Mountains Eye Study^16^ reported the prevalence of thyroid dysfunction in men aged 80+ years was 4.3%, similar to the 3.5% prevalence of minor thyroid dysfunction of men aged ≥75 years in the Whickham^17^ study and 1.5% in men ≥73 years in a Dutch study.^18^ Our findings are more closely aligned with those of the Colorado study^19^ that reported a prevalence of thyroid dysfunction in men ≥75 years of 16% and another study in which the prevalence of thyroid dysfunction in men aged 85 years was 10.7%.^20^ However, for some of these studies the study population sizes looking purely at the eldest age stratum of men was small, with the Whickham study looking only at 57 such men and the Blue Mountains Eye Study analysing 94 such men.^16^, ^17^ As a result, those studies may have lacked the power to accurately define the prevalence of thyroid dysfunction in this understudied demographic group. We found that thyroid dysfunction is highly prevalent in the oldest stratum of men.

Our data show a progressive increase in TSH over 8 years, across the range of TSH values. By contrast, change in FT4 was inversely correlated with baseline FT4 in a pattern consistent with regression to the mean, with little overall change in FT4 concentrations. Further analysis demonstrated that in those who remained euthyroid, both FT4 and TSH increased to a small extent whereas in those who developed subclinical hypothyroidism, TSH increased fivefold more than in those who remained euthyroid, but FT4 was unchanged. These data suggest that our study sample consisted of two groups. The larger group comprised those who remained euthyroid and in whom there was an age‐related increase in the TSH‐FT4 set‐point. The men in the smaller group appeared to have evolving thyroid dysfunction as the age‐related increase in TSH was larger and was not accompanied by an increase in FT4.

Several cross‐sectional studies of community‐representative cohorts reported an increase in TSH with age in men.^21^, ^22^ Longitudinal studies of changes in thyroid function in euthyroid middle‐aged and older adults demonstrated a within‐individual increase in TSH that was not accompanied by a fall in FT4 concentrations, suggesting an age‐related alteration in set‐point of the pituitary–thyroid axis, rather than an increased prevalence of occult thyroid disease.^10^, ^11^ This change in the set‐point may be evident from the attenuated increase in nocturnal TSH pulse amplitude reported in older compared with younger males despite higher baseline TSH concentrations.^23^ The results from the men in our study who remained euthyroid are consistent with this hypothesis.^24^ In addition, given that both FT4 and TSH increased slightly in those who remained euthyroid, our results are more consistent with explanations that involve diminished pituitary sensitivity to thyroid hormone feedback, rather than diminished thyroid responsiveness to TSH or diminished TSH bioactivity with age, but further research is needed to distinguish these possibilities.

We demonstrated an exponential increase in the risk of subclinical or overt hypothyroidism over 8 years as baseline TSH increased. Whereas other authors examining this question have identified TSH break‐points above which risk seems to increase progressively^25^, ^26^ our data suggest there is no clear cut‐point and this is more in keeping with the data of Asvold et al.^27^ Our ROC analysis indicated a TSH cut‐point at 2.34 mU/L would have optimal sensitivity and specificity for predicting future hypothyroidism. This is consistent with the value of 2.3 mU/L identified as the optimal threshold from ROC analysis for risk of future hypothyroidism in men in the Busselton study^26^ and similar to the values of 2.0 and 2.6 mU/L identified for women in the Whickham and Busselton studies.^25^, ^26^ Our results also identified that euthyroid men with TSH ≥ 2.85 mU/L have >20% chance of developing hypothyroidism over 8 years, suggesting that closer monitoring for thyroid disease should be considered in men with TSH results in this range. The probability for hypothyroidism at any given TSH value appears to be higher than for the participants in other studies,^25^, ^26^, ^27^ most likely because of the older age of the men in our study.

The US Preventative Services Task Force published a comprehensive evaluation of population screening for thyroid disease in 2015,^28^ finding that ‘the current evidence is insufficient to assess the balance of benefits and harms of screening for thyroid dysfunction in nonpregnant asymptomatic adults’. This was based upon the lack of evidence that screening could produce clinically significant changes in BMI, blood pressure, bone mineral density or lipid profile. An evidence review in 2021 did not change this conclusion.^29^ The UK NICE guideline for assessment and management of thyroid disease also suggests that thyroid function testing be reserved in adults with a clinical suspicion of thyroid dysfunction or for patients with type 1 diabetes, atrial fibrillation or psychiatric disorders.^30^ Within Australia, recent recommendations do not specifically address the question of whether asymptomatic patients should be screened, instead focussing on diagnosing hypothyroidism appropriately in those with symptoms, which are often mild and nonspecific.^31^

Overt hypothyroidism has been found to have profound effects on cardiovascular health, however, subclinical hypothyroidism has remained more controversial as a predictor of cardiovascular disease.^24^ Recent meta‐analyses of epidemiological studies have produced inconsistent results, particularly regarding the association between subclinical hypothyroidism and all‐cause mortality.^32^, ^33^ This issue assumes greater importance for older men. Our study found that a third of older men who had subclinical hypothyroidism progressed to becoming overtly hypothyroid. Higher TSH values were associated with a greater risk of developing thyroid dysfunction and of those who were initially euthyroid, men with a TSH > 2.85 mU/L had more than a 20% chance of developing subclinical or overt hypothyroidism over a 9‐year period. These findings suggest that further research is required to examine the utility of systematic screening of thyroid function tests in the older male population, particularly for those with elevated baseline TSH values.

The strengths of this study include the large community‐based nature of the cohort, the detailed characterisation of the men and the longitudinal nature of the study. FT4 and TSH concentrations were measured in participants at both timepoints. The focus on the stratum of oldest old men (mean age 75.9 years) is informative as these men have higher rates of progression to thyroid disease. Men in this study were community‐dwelling and voluntarily visited a collection centre for venesection, suggesting that these men were not acutely unwell and making the presence of sick euthyroid syndrome less likely. We acknowledge several limitations of this work. HIMS is an observational study. Many variables were obtained by self‐report. No measure of iodine intake as a variable was collected in HIMS. However, Australian health statistics indicate the majority of older men within Australia are iodine‐sufficient.^34^ Furthermore, TPO antibodies were not assessed at Wave 2; thus, we cannot analyse longitudinal changes in TPO antibodies between Wave 2 and Wave 4, nor can we comment on a possible causative effect of TPO positivity on incident thyroid dysfunction. Men included in this analysis were drawn from a larger sample of 19,352 men surveyed previously and hence, a ‘healthy survivor’ effect may be present. As a result, our findings may be most applicable to healthier community dwelling elderly men. The study population is primarily of White ethnicity and findings from our current analysis might not apply to men from other ethnic backgrounds or younger men and we cannot comment on changes in thyroid function in women. Analysis of younger men, or of women, was outside the scope of this study. The probability calculations used an estimate of prior probability of becoming hypothyroid derived from this sample of men, which may therefore limit the comparability of our results to men in other settings and to women. The TSH threshold for >20% risk of developing hypothyroidism is not a threshold for initiating treatment and our data do not provide evidence in support of the initiation of therapy in subclinical hypothyroidism.

In conclusion, in older men, TSH increases over time with the largest increments seen in men with high‐normal TSH at baseline, whilst changes in FT4 were modest and consistent with regression to the mean. In men who remained euthyroid, both TSH and FT4 increased modestly. However, men who developed subclinical hypothyroidism had larger increases in TSH while FT4 remained unchanged, suggesting separate populations of men remaining euthyroid and becoming hypothyroid. Age and higher baseline TSH predict the evolution of subclinical or overt hypothyroidism, which occurs in a substantial proportion of older men. The risk of becoming hypothyroid over 8 years increased to over 20% in those with baseline TSH > 2.85 mU/L, which may be a useful threshold to consider increased thyroid surveillance in older men. Additional research is warranted to determine whether active case‐finding for subclinical or overt hypothyroidism would be beneficial in the expanding demographic of older men.

## CONFLICT OF INTEREST STATEMENT

The authors declare no conflict of interest.

## Data Availability

Restrictions apply to the availability of some or all data generated or analysed during this study, to preserve participant confidentiality. The corresponding author will on request detail the restrictions and any conditions under which access to some data may be provided.
